# Radioactive Springs and Archaeal Life in Deep Groundwater Systems

**DOI:** 10.1007/s00248-026-02720-7

**Published:** 2026-03-14

**Authors:** Terézia Eckertová, Andrea Palyzová, Monika Műllerová, Tomáš Řezanka

**Affiliations:** 1 Department of Nuclear Physics and Biophysics, Faculty of Mathematics, Physics and Informatics, Comenius University in Bratislava, Mlynská Dolina F-1, Bratislava, 841 04 Slovak Republic; 2 Institute of Microbiology, Czech Academy of Sciences, Vídeňská 1083, Prague, 142 00 Czech Republic

**Keywords:** Archaea, Shotgun lipidomics, Mass spectrometry, Radioactive springs, Microbial communities, Groundwater ecology

## Abstract

This study investigates the archaeal lipid distribution in freshwater springs with a particular focus on lipidomic profiles as ecological indicaters. Cultivation-independent approaches were employed to analyze organisms that had not yet been cultivated in the laboratory. Shotgun lipidomics of 21 springs in western and central part of Slovakia revealed more than 100 characteristic archaeal lipids, from which three biomarker groups were selected: (i) core lipids containing archaeol and glycerol dialkyl glycerol tetraethers (GDGT), including their mono and dihydroxy derivatives; (ii) mono- to tetra-glycosides of archaeol and GDGTs; and (iii) six phosphoarchaeols (archaeol-based phospholipids). Statistical analyses classified springs into three categories: cold (temperature < 20 °C), warm (> 30 °C), and radioactive (a subset of cold springs with ˃100 Bq/L radioactivity). Significant shifts in the ratios of archaeal lipids were correlated with the temperature and radioactivity, demonstrating the sensitivity of lipidomic profiling to environmental parameters. Moreover, tandem mass spectrometry identified a previously undescribed metabolite, archaeol-based dimethylphosphatidylethanolamine. The applied method provides rapid and highly sensitive tools for screening the presence of archaea, detecting as few as several thousand cells per liter, and offers new insights into the ecology of archaeal communities in groundwater environments.

**Supplementary Information:**

The online version contains supplementary material available at 10.1007/s00248-026-02720-7.

## Introduction

The natural radioactivity of groundwater and surface waters is controlled by the occurrence of dissolved natural radionuclides, mainly potassium (^40^K), radon (^222^Rn), radium (^226^Ra), thorium (^232^Th), and uranium (^234^U, ^235^U, and ^238^U). The transfer of radionuclides from rocks to water occurs through water-rock interactions and proceeds primarily through dissolution, where radionuclides are released by disrupting the crystal lattice, and leaching, whereby radionuclides are mobilized without lattice damage [1, 2]. Dissolution produces water compositions that broadly reflect those of the host rock, while the mode of incorporation into minerals strongly influences leaching. Daughter products are mobilized from microcapillaries and are often crystal defects, rarely stabilized as independent mineral phases. Consequently, leaching represents the main pathway of radionuclide enrichment, resulting in the predominant accumulation of decay products in natural waters [1, 3].

Radon (^222^Rn), a short-lived noble gas generated by the decay of ^226^Ra, represents a key contributor to the natural radioactivity of groundwater. Radon can remain within mineral structures or accumulate in pores and fractures. Its transfer to groundwater is governed primarily by emanation from mineral surfaces, while diffusion through the solid matrix is negligible [4]. High concentrations of radon are generally associated with fractured crystalline rocks and tectonic fault zones, where groundwater circulation improves enrichment [5, 6]. Although radon is highly soluble, its solubility decreases with increasing temperature. Dissolved radon can be classified as allogenic, subdivided into hyperallogenic (derived from the weathering crust) and hypoallogenic (originating from deeper fault zones), or autigenic, formed in situ by decay of radium dissolved in water [1, 5].

Radon-rich waters are characteristic of crystalline terrains, particularly granitoids and migmatites, where fractured weathering zones host springs with elevated radon activity [7] In Central Europe, such waters occur in the spa regions of Jáchymov and Jánské Lázně (Czech Republic), Bad Brambach and Bad Schlema (Germany), and Lądek-Zdrój (Poland), where the radon activities exceed several kBq/L [7–10].

In Slovakia, radon-rich springs are widespread, reflecting the complex geological setting of the Western Carpathians [11]. They occur in association with granitoid bedrock, crystalline schists, travertine accumulations, and tectonically active fault zones [12]. Several categories of radon-bearing waters have been identified, including springs emerging along tectonic zones and travertine deposits, such as those in the Vyšné Ružbachy and Číž–Tornaľa regions [13]. Distinct hydrogeochemical types of radon-rich groundwater include waters related to clay–travertine sediments enriched in radium, thermally elevated waters associated with deep tectonic faults [5] and radon-bearing waters linked to uranium ore bodies [1]. These fluids often ascend along fault zones of varying depth and mineralogy, carrying elevated concentrations of radium and dissolved CO_2_, with radon coming from both deep geological reservoirs and radium deposits on fracture surfaces [6].

Additionally, springs near Piešťany discharge from Triassic carbonate aquifers and have been used for balneotherapy for a long time [14, 15]. Recent Slovak studies have increasingly focused on hydrogeological characterization and radiological safety, advancing the understanding of radon migration within geological media [13, 16].

Beyond their radiological features, these deep subterranean springs are valuable ecosystems that host microbial communities adapted to extreme conditions: low nutrients, high pressure, and limited organic carbon [17–20]. These microorganisms use inorganic electron donors, such as hydrogen, reduced sulphur compounds, and ferrous iron, for biomethane production. This makes them ecologically significant and potentially relevant to securing new sources of renewable energy in the field of biotechnology [21]. In particular, bacteria and methanogenic archaea form syntrophic consortia, contributing to the production of methane in these oligotrophic settings [22, 23]. Such findings have implications not only for groundwater ecology but also for understanding microbial life in subsurface environments in other locations on the Earth, including deep-sea hydrothermal vents [24].

**Fig. 1:**
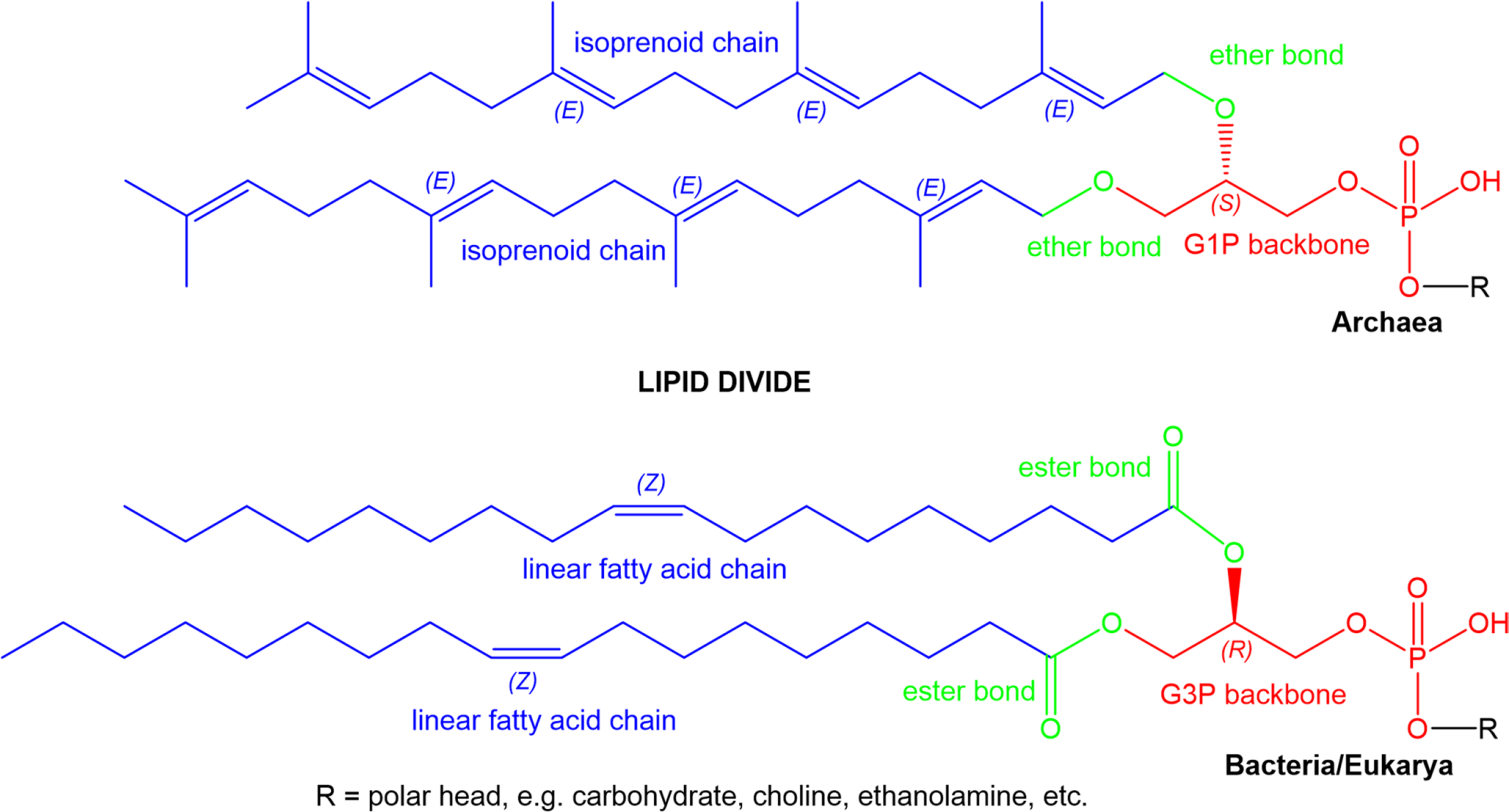
The basic skeletal structure of phospholipids in archaea and bacteria/eukarya; three core dimensions are marked with different colors

A key molecular characteristic of archaeal adaptation is its unique membrane lipid composition, which distinguishes them from bacteria and eukaryotes (Fig. 1) [25, 26]. Archaeal membranes are composed of ether-linked isoprenoid chains bound to *sn*-glycerol-1-phosphate (G1P), in contrast to ester-linked fatty acids in *sn*-glycerol-3-phosphate (G3P) found in other domains of life [27]. This structural divergence, central to the hypothesis of the “lipid divide“, likely reflects early evolutionary pathways from a last universal common ancestor (LUCA) not bound to the membrane [28]. Archaeal ether lipids provide chemical and thermal resilience, enabling survival in extreme environments such as hydrothermal vents and radon-rich springs.

The simplest archaeal lipid, archaeol, consists of two phytanyl chains, while more complex variants include extended or cyclized forms and modifications of the polar head group (e.g., phosphatidylinositol, glycosylated derivatives) [29]. Even more thermostable are glycerol dialkyl glycerol tetraethers, which form monolayer membranes and can contain up to eight cyclopentane rings [30]. GDGTs, especially those produced by thermophilic and methanogenic archaea, are widely used as biomarkers in paleoenvironmental studies (e.g., TEX86 indices) [31].

Modern high-resolution mass spectrometry (HRMS) allows routine analysis of hundreds of lipid species in complex mixtures such as biological samples, but rapid technological advances have led to a dramatic increase in the complexity and size of data sets due to higher mass and chromatographic resolution, faster data acquisition, more sophisticated workflows, and combinations of techniques. Improved mass spectrometer designs, including more powerful computers, now enable the collection of more data, thus increase the number of detected signals. These advances in HRMS, including approaches such as shotgun lipidomics or HRMS coupled with chromatographic separation (LC-MS), allow detailed profiling of archaeal lipids from complex environmental samples [32], which is crucial to studying the diversity, ecological roles, and evolutionary history of archaea in subsurface ecosystems, including Slovak radon aquifers.

Based on statistical analysis of the relevant biomarkers of archaeal lipids, including core lipids and intact polar lipids, shotgun lipidomics can clearly distinguish individual types of spring sources and enable rapid screening for archaeal presence with a sensitivity sufficient to detect as few as a few thousand cells per liter of water.

This study aims to determine how temperature and radon-derived radioactivity influence the archaeal lipidome in deep groundwater springs and to identify lipid biomarkers that reflect these specific environmental conditions. We focused on the characterization of membrane lipid profiles in response to extreme environmental conditions. Using multivariate analysis, we demonstrate that the archaeal lipid profiles differ markedly between radon-rich cold springs, non-radioactive cold springs, and thermally hot springs.

## Materials and Methods

### Collections of Water and Extraction of Lipids

For the analysis of total lipids in each spring (see Table S1), two 1.5 L sterile bottles were filled with water, and the temperature of the water was measured. The water from 21 springs (see Table S1, Supplements) was filtered by 0.11 μm membrane filters (VWR, USA). Subsequently, all filters were extracted according to Bligh and Dyer [33] a mixture of methanol (MeOH)/dichloromethane (DCM)/phosphate buffer (pH 7.4; 2:1:0.8, v/v/v) in an ultrasonic bath (2 × 15 min). Due to the differing hydrophobic properties of the lipid classes, a complete lipid profile could not be obtained with a single extraction procedure. Therefore, two successive extractions were performed according to Markham and Jaworski [34]. The first was a classical extraction with a methanol-dichloromethane mixture. The second extraction is an isopropanol-hexane-water mixture using a mixture of isopropanol/hexane/water (55:20:25 v/v/v), which allows the extraction of even very polar lipids, such as oligoglycosidic derivatives (up to GDGT tetrasaccharide glycosides). The supernatants were combined, and DCM and water were added. The DCM phase was collected and dried under the flow of N_2_.

For measuring radon activity concentration (RAC) in water, we used a scintillation chamber (Lucas cell, volume 125 mL) and a scintillation probe, both supplied by the EMPOS company, connected within a CAMAC electronic system. Air was drawn through 7 mL of the water sample into the evacuated chamber, transferring the dissolved radon to the Lucas cell. Each water sample was transferred to the chamber twice, each measurement lasting one hour. The final RAC value was calculated as the mean of the two measurements.

### Shotgun Analysis

The linear ion trap quadrupole-LTQ-Orbitrap (LTQ-Orbitrap) Velos mass spectrometer (Thermo Fisher Scientific, San Jose, CA, USA) was equipped with a heated electrospray interface (HESI) in positive and negative ionization modes. The mass spectrometer scan range was performed on the Fourier transform (FT) cell and recorded within a window of 200–2000 Da. The mass resolution was set at 105,000, and the ion spray voltage was + 3.5 kV in the positive ionization mode. The ionization mode used the following parameters: N_2_ was used as a nebulizer gas and set at 18 arbitrary units (sheath gas) and 7 arbitrary units (auxiliary gas); ion source temperature, 250 °C; capillary temperature, 230 °C; capillary voltage, 50 V; and tube lens voltage, 170 V. Helium was used as a collision gas for collision-induced dissociation (CID) experiments. The CID normalization energy of 35% was used to fragment the parent ions. Tandem MS (MS/MS) product ions were detected in a high-resolution FT mode.

The mass spectrometer was calibrated using a Pierce LTQ Orbitrap positive-ion calibration solution (Thermo Fisher Scientific, San Jose, CA, USA). For mass spectral acquisition, the internal lock mass was used: *m/z* 413.2662 [M + Na]^+^ i.e., diisooctyl phthalate in ESI^+^. The mass accuracy was better than 1.0 ppm. The core lipid structure and also intact polar lipids were confirmed with the help of the LIPID MAPS Lipidomics Gateway spectral database (LIPID MAPS, 2023) and previously published descriptions of positive mass spectra of core lipids and intact polar lipids (IPL) [35–37].

The metabolic potential of archaeal genome assemblies was determined by mapping pathway information to the resulting functional annotations, which were generated using KEGG profiles (https://www.genome.jp/kegg/pathway.html). These predicted functions were then compared with the lipid metabolites detected in the sediments, in order to evaluate whether the identified pathways could account for the observed archaeal lipid signatures.

### Calibration

The commercially available archaeol standard 4ME 16:0 diether DG (1,2-di-O-phytanyl-sn-glycerol) was purchased from Merck (Prague, Czech Republic) and analyzed separately. The archaeol stock standard was stored at -20 °C. The ion at *m/z* 373.3676 (from PIS) was used to generate a calibration curve using the peak area of the tandem MS corresponding to the different concentrations of the standard (1.0, 2.0, 5.0, 10.0, 20.0, 50.0, 100, 200, and 500 femtomol/µL, 1, 2, 5, 10, 20, and 50 picomol/µL). The linear regression of the calibration curve was calculated, as well as the signal/noise (S/*N* = 3) ratio. The assays were linear throughout the concentration range, ranging from 5 pmol/µL to 20 nanomol/µL, with a mean determination coefficient R^2^ of 0.9914. The third point of the calibration curve (5.0 femtomol/µL) corresponds to the lower limit of quantification (LLOQ). The detection limit (LOD) for achaeol was determined and the concentration was 2.0 femtomol/µL, which is approximately 1.3 picog/µL (exact value 1306 femtog/µL). Other chemicals used were also purchased from Merck.

### Statistical Analysis

For the statistical evaluation of archaeal lipid profiles from 21 springs, a multivariate analysis approach was applied using two complementary software platforms: PAST v5.3 and OriginPro 2024 (OriginLab Corporation, MA, USA). Principal Component Analysis (PCA) and hierarchical cluster analysis (HCA) were conducted in OriginPro. PERMANOVA (one-way, pairwise, and two-way), SIMPER, Redundancy Analysis (RDA), and Partial Least Squares (PLS) were performed in PAST. Variable Importance in Projection (VIP) scores were subsequently obtained in OriginPro.

## Results

### Shotgun Lipidomic Analysis

The total lipid extracts were analyzed by shotgun lipidomics using HRMS with positive ESI on an Orbitrap instrument (see Materials and Methods). The distribution of individual lipids, comprising both core lipids and IPL, is summarized in Table S2. A total of 102 molecular species were identified. The core lipids were dominated by archaeol (AR) and its monohydroxy and dihydroxy derivatives, together with GDGT containing up to five cyclopentane rings. In contrast, IPLs exhibited greater structural diversity, primarily as phospholipid derivatives of AR and glycosylated forms ranging from mono- to trihexosides, including monounsaturated triglycosyl archaeol.

### Multivariate Analysis of Environmentally Structured Archaeal Lipids in Springs

As noted above, 102 molecular species from 21 sources were analyzed using shotgun lipidomics. Tandem mass spectrometry in positive ionization mode was used to identify both core lipids, phospholipids, and glycolipids, as it provides information on the structural characteristics of isoprenoid chains. To better resolve the relationships among the types of springs (cold, warm, or characterized by elevated radioactivity), three groups of biomarkers were selected: core lipids, archaeol phospholipids, and archaeol glycolipids. The HCA dendrograms consistently suggested the existence of three main spring clusters, indicating that the distribution of archaeal biomarkers is not random but structured according to environmental characteristics (Fig. S1).

**Fig. 2:**
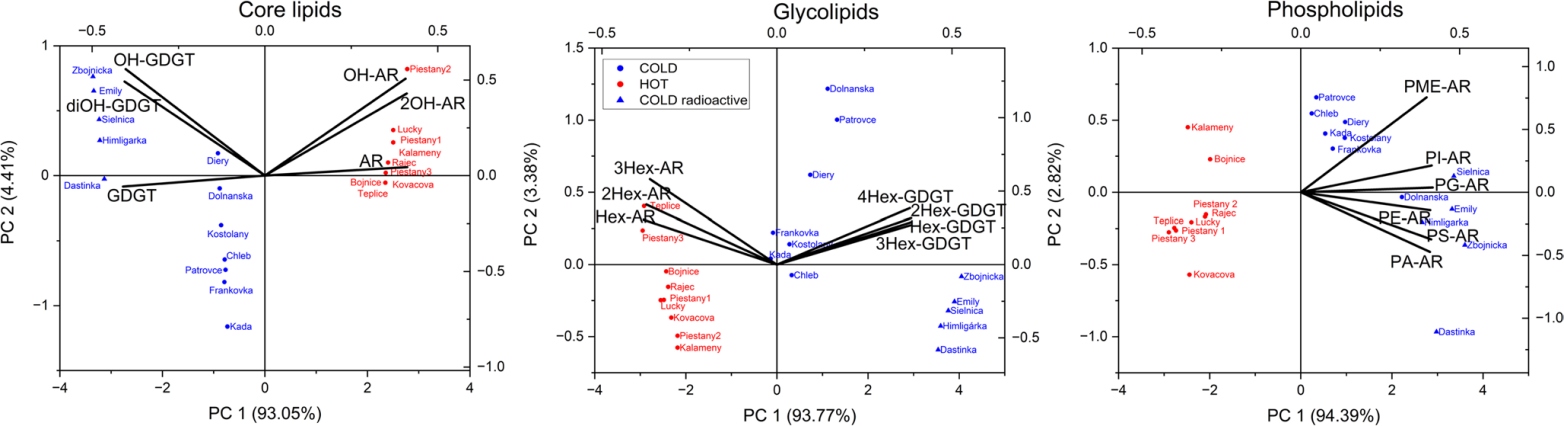
Principal component analysis (PCA) of archaeal lipid biomarkers in 21 springs, showing separation of samples based on core lipids, glycolipids, and phospholipids

The principal component analysis (PCA), see (Fig. 2), was applied to the values presented in the relevant tables. The PCA plots revealed a clear separation of the samples along the first component, which represented the majority of the variance in all three lipid classes. The loading vectors of individual biomarkers highlighted the compounds that drive this differentiation. In particular, PCA indicated that both temperature and radioactivity contribute to the structuring of archaeal lipid assemblages.

**Fig. 3:**
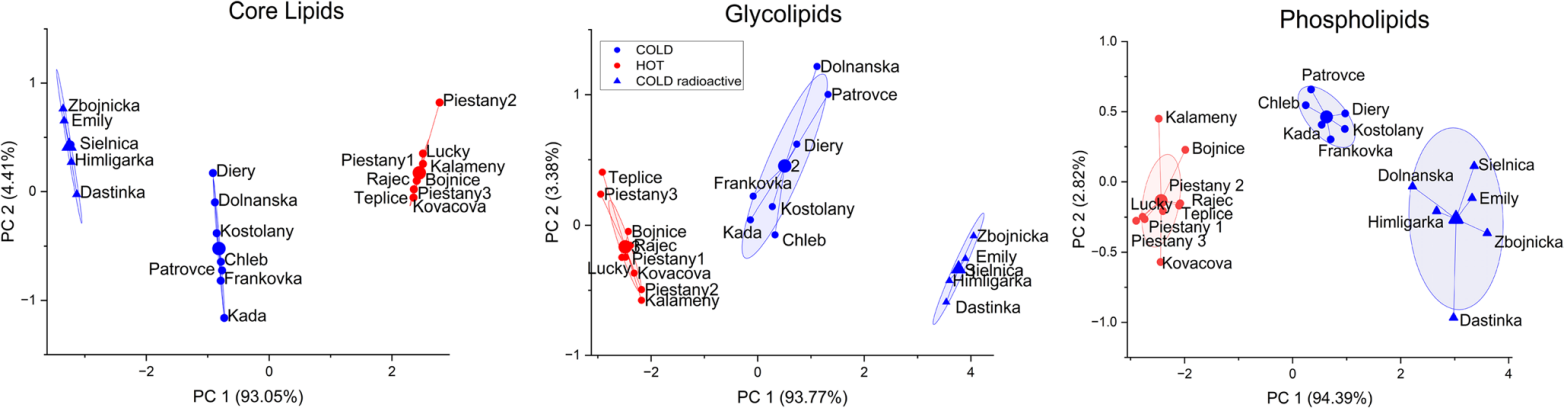
K-means cluster analysis of archaeal lipid profiles, illustrating three major spring categories: cold, warm, and radioactive cold springs

Cluster analysis (Fig. 3) provided an even more explicit visualization of this pattern, confirming the classification of the springs into three distinct categories: (a) cold springs, (b) warm springs, and (c) radioactive cold springs. Strong coherence and sharp separation between groups suggest that archaeal communities are shaped by the combined influence of thermal and geochemical factors. In particular, the radioactive springs formed a coherent cluster different from the other cold waters, supporting the hypothesis that radioactivity imposes selective pressure on archaeal populations.

The visual distributions for individual compounds are presented in Fig. S2, with supporting values in Tables S3, S4, and S5. These results corroborate the data of multivariate analyses, indicating that specific biomarkers are enriched in particular spring categories.

To complement these exploratory analyses, PERMANOVA confirmed significant differences in archaeal lipid composition among the three spring categories: radioactive cold vs. cold (pseudo-F = 42.7, *p* = 0.0012), radioactive cold vs. warm (pseudo-F = 605.5, *p* = 0.0003), and cold vs. warm (pseudo-F = 102.4, *p* = 0.0002). A two-way PERMANOVA further showed that both temperature (pseudo-F = 191.93, *p* = 0.0001) and radioactivity (pseudo-F = 161.42, *p* = 0.0001) independently contribute to lipid variability, with no significant interaction between these factors. SIMPER analysis identified GDGTs (GDGT, OH-GDGT, diOH-GDGT) as the dominant contributors to dissimilarity across spring types, while 2OH-AR and OH-AR were key for differentiating cold (non-radioactive) from warm springs. Redundancy analysis (RDA) supported these patterns, revealing that a single dominant environmental gradient – combining radon activity and temperature – explains most of the variance (R2 = 0.884) in lipid profiles. Finally, PLS analysis highlighted five compounds with the highest discriminatory power (VIP > 1.10): PME-AR, 3Hex-AR, 2OH-AR, diOH-GDGT, and OH-AR. Loadings further indicated that GDGT-based lipids are most associated with radioactive cold springs, whereas PME-AR and 3Hex-AR best characterize warm springs. Complete VIP scores and loading values are provided in Supplementary Table S7.

### Identification of DMPE in Biomass from Chleb and Sielnica Springs

**Fig. 4:**
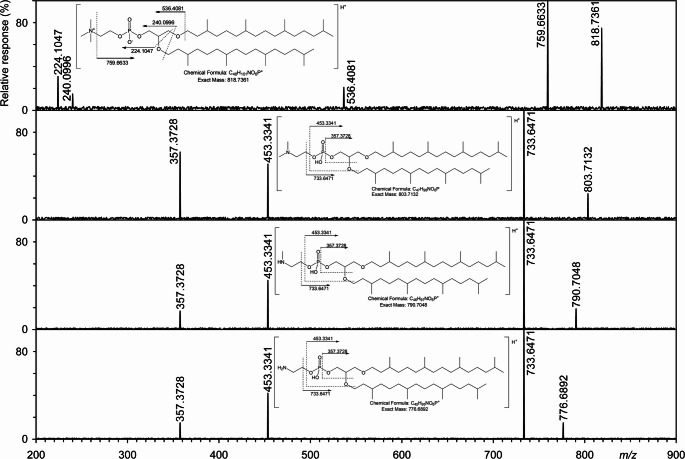
Negative tandem high-resolution mass spectrum of the major molecular species of PE-AR, MMPE-AR, DMPE-AR, and PC-AR. DMPE-AR (2,3-bis((3,7,11,15-tetramethylhexadecyl)oxy)propyl (2-(dimethylamino)ethyl) hydrogen phosphate) was isolated from only two sources (Chleb and Sielnica), see Table S1. A detailed description of the ions is in the text, see subsection “Identification of DMPE in biomass from Chleb and Sielnica springs”

Tandem MS in positive ionization mode was used to identify both core lipids, phospholipids, and glycolipids, as it provides information on the structural characteristics of isoprenoid chains; see Discussion. The molecular IPL ions obtained in the positive mode were observed in their protonated form ([M + H]^+^). During the neutral cleavage of alkyl substituents in MS/MS, an H atom is transferred from the phytanyl chain. For example, in AR, the saturated phytanyl substituent is split as phytene (280.3130 Da). In tandem MS, saturated phospholipids, such as those shown in DMPE-AR (Fig. S3), partially lose the head group while retaining phosphatidic acid (PA), as shown by the main peak (base peak) at *m/z* 733.6471. In MS/MS experiments, saturated phospholipids provide a main peak corresponding to neutral loss of the head group (Fig. 3), (e.g. 43.0422 Da PE, 57.0578 MMPE, 71.0735 DMPE), while retaining phosphatidic acid, which shows a major peak at *m/z* 733.6471 (PA-AR). The ions of the less abundant fragments, especially at *m/z* 357.3728 and *m/z* 453.3341 (Fig. 3), are the result of the loss of a phytanyl group (like phytene, loss of 280.3130 Da) from the main ions in MS/MS Fig. 4). DMPE-AR also gives a less abundant ion of type [M + H]^+^ at 803.7132 Da with the general formula C_47_H_98_NO_6_P.

## Discussion

Tandem MS in positive ionization mode was used to identify both core lipids and phospholipids and glycolipids, as it provides information on the structural characteristics of isoprenoid chains, see [38]. Archaea are a crucial component of microbial communities on modern Earth and are particularly abundant in extreme environments, such as spring systems [39, 40]. Therefore, archaeol or caldarchaeol are often used as biomarkers of the presence of Archaea in waters [41–43]. Both lipids, i.e., core lipids and IPLs, were used for taxonomic purposes, both in Japan Sea sediments [44] and the Black Sea [28]. Comparing the results in Table S2 with published data, it was identified that glycolipids up to 5Gly-AR or 4Gly-GDGT are present in deep-sea hydrothermal vents (Mid-Atlantic Ridge) [45].

Principal component analysis has been used several times for GDGT or glycerol dialkyl diethers (GDDs) to distribute as core lipids (Lake Chala and Azorean lakes, including sediments of the Mediterranean, Black, and Arabian Sea) [45] as well as core and polar lipids (hot springs in Tibet) [46, 47] statistically compared data in several dozen articles already published. This was mainly a temperature index based on GDGT (TEX_86_) with two and three cyclopentane rings (GDGT-2/GDGT-3). To the best of our knowledge, no studies published to date have conducted a comprehensive comparative analysis of the full spectrum of archaeal lipids, which encompasses both core lipids and intact polar lipids containing AR and GDGT moieties as alcohol backbones, along with their homologues, hydroxylated derivatives, and variants differing in the number of unsaturations or cyclopentane rings. Moreover, the use of IPL-based approaches for rapid detection and characterization of archaeal communities in freshwater ecosystems remains largely unknown, particularly in cold springs enriched in natural radionuclides.

Derivatives of phosphatidylethanolamines, diacyl-PE, mono-, di-, and trimethyl-phosphatidylethanolamines (with the latter corresponding to phosphatidylcholines) have been described in a wide variety of organisms, ranging from bacteria [48–50] to mammals [51]. Table S2 lists nitrogenous derivatives of intact polar lipids, including three homologues of nonhydroxylated archaeol-based derivatives, namely PE-AR, PME-AR, and DME-AR. In addition, three hydroxylated counterparts (PE-OH-AR, PME-OH-AR, and DME-OH-AR) were detected in waters from mineral springs located in Slovakia. Similar compounds have been reported from other geographically distant environments, including freshwater systems such as the White Oak River Basin and a variety of marine sediments, notably those from the Wadden Sea, Mediterranean Sea Black Sea, and the Peru margin [52]. The presence of PE-AR has also been documented in the *Methanosarcina barkeri* [53]. Furthermore, PE-AR and PME-AR have been identified in Makran cold seeps and Alaskan lakes [54], where both phosphatidylethanolamine-archaeol (PE-AR) and phosphatidylcholine-archaeol (PC-AR) were observed.

In two of the 21 springs analyzed in the western and central part of Slovakia, dimethylphosphatidylethanolamine-archaeol (DMPE) was identified. This lipid has not yet been described in natural environments nor synthesized in the laboratory. Both springs are characterized by low water temperatures (~ 5 °C) and high-altitude settings (approximately 1400 m at Chleb and 990 m at Sielnica; Table S1). This suggests that Archaea inhabiting these cold aquatic environments incorporate DMPE into their membranes to ensure optimal fluidity under low-temperature conditions. Given the low temperature, negligible salinity, and the contrast with deep-sea hydrothermal systems that typically host thermophilic archaea, these springs may contain highly specialized and probably previously undescribed archaeal lineages.

Analysis of the KEGG database indicates that more than 30 archaeal species are putatively represented by two key *N*-methyltransferases (EC 2.1.1.17 and EC 2.1.1.71), which catalyze successive methylation of the amino group of ethanolamine to produce the trimethylethanolamine moiety. These enzymatic transformations are well documented in both bacterial and eukaryotic systems [55]. Similar results of the Kegg database are summarized in Table S6, and three representative examples are provided in Figures S4–S6. Based on the occurrence of the three metabolites in sediments and the proposed biosynthetic pathway, the presence of DMPE-AR in our samples is highly probable. Assuming that phosphatidylcholine-archaeol is synthesized from PE-AR by sequential methylation of the amino group, DMPE-AR would represent a missing intermediate in this pathway. Consistent with this hypothesis, PE-AR, monomethyl-PE-AR (MMPE-AR), and PC-AR have been previously described.

To identify robust biomarkers for Archaea, we evaluated more than 100 known archaeal lipids and selected three main groups: (i) archaeol and glyceroldialkylglycerol tetraethers, including their monohydroxy and dihydroxy derivatives; (ii) archaeol and GDGT-based mono- to tetraglycosides; and (iii) six distinct phospho-archaeols (phospholipids derived from archaeols). Statistical evaluation of these biomarkers confirmed that the springs can be classified according to temperature and radioactivity into three categories: cold springs, warm springs, and radioactive springs.

The analytical approach used here provides a rapid and sensitive means of detecting archaeal signatures in natural waters. As demonstrated by [31] this method achieves sufficient sensitivity to detect archaeal populations as small as a few thousand cells per liter of water, making it particularly suitable for screening of microbial life in extreme or poorly characterized environments.

## Conclusion

A comprehensive analysis of archaeal core and intact polar lipids revealed a significant ecological differentiation in archaeal communities across freshwater springs. The detection of DMPE, a previously unreported compound, suggests that the composition of cell membranes is adapted to low-temperature and loe salinity conditions, and indicates the presence of highly specialised, potentially undescribed archaeal lineages. The classification of springs according to their environmental using lipid-based biomarker patterns highlights the value of intact polar lipid approaches for microbial ecological investigations in extreme and poorly characterised freshwater ecosystems.

### Data Availability

The data supporting this study are available in Zenodo under 10.5281/zenodo.17853821.

## Supplementary Information

Below is the link to the electronic supplementary material.

Supplementary Material 1 (DOCX 1.91 M)

## Data Availability

The data supporting this study are available in Zenodo under https://doi.org/10.5281/zenodo.17853821.

## Abbreviations

2Hex-AR: dihexosyl-archaeol
2Hex-GDGT: dihexosyl-glycerol dialkyl glycerol tetraether
2OH-AR: dihydroxy-AR
3Hex-AR: trihexosyl-archaeol
3Hex-GDGT: trihexosyl-glycerol dialkyl glycerol tetraether
4Hex-GDGT: tetrahexosyl-glycerol dialkyl glycerol tetraether
AR: archaeol
diOH-GDGT: dihydroxy-GDGT
GDGT: glycerol dialkyl glycerol tetraether
Hex-AR: hexosyl-archaeol
Hex-GDGT: hexosyl-glycerol dialkyl glycerol tetraether
MMPE-AR: monomethylphosphatidylethanolamine-AR
OH-AR: hydroxy-AR
OH-GDGT: hydroxy-GDGT
PA-AR: phosphate-AR
PC-AR: phosphatidylcholine-AR
PE-AR: phosphatidylethanolamine-AR
PG-AR: phosphatidylglycerol-AR
PI-AR: phosphatidylinositol-AR
PS-AR: phosphatidylserine-AR
