# Supplementary material for: Radioactive Springs and Archaeal Life in Deep Groundwater Systems
Source: Microb Ecol. 2026 Mar 14;89(1):83. doi: 10.1007/s00248-026-02720-7 (PMC13032954; doi:10.1007/s00248-026-02720-7)
Supplement: Supplementary file 1 — Supplementary Material 1 (DOCX 1.91 M) [file 248_2026_2720_MOESM1_ESM.docx]

**Supplements**

**Radioactive Springs and Archaeal Life in Deep Groundwater Systems of Slovakia**

Terézia Eckertová^a^, Andrea Palyzová^b^, Monika Műllerová^a^, Tomáš Řezanka^b,*^

^a^ Department of Nuclear Physics and Biophysics, Faculty of Mathematics, Physics and Informatics, Comenius University in Bratislava, Mlynská Dolina F-1, 841 04 Bratislava, Slovak Republic

^b^ Institute of Microbiology, Czech Academy of Sciences, Vídeňská 1083, 142 00 Prague, Czech Republic

**Table S1.** Information about 21 springs, including their location, date of sampling and filtration together with filtered volume of water, number of used filters, temperature, flow rate, and radon activity concentration (RAC).

| **Sample^a^** | **GPS (°)** | **Sampling** |  | **Filtration** | **Filtered volume (mL)** | **No. of filters** | **Temperature (°C)** | **Flow rate (L/min)** | **RAC (Bq/L)** |
| --- | --- | --- | --- | --- | --- | --- | --- | --- | --- |
| Dastinka | 48.643743-17.9736137 | 10.6.2025 9:30 |  | 11.6.2025 | 3070 | 2 | 12.8 | 1.5 | 203.7 ± 8.2 |
| Emily | 48.3150278-17.2027778 | 17.6.2025 10:05 |  | 17.6.2025 | 3140 | 1 | 9.4 | 6.9 | 277.3 ± 16.1 |
| Himligarka | 48.2336881-17.1266469 | 18.6.2025 9:20 |  | 18.6.2025 | 3140 | 1 | 9.1 | 1.4 | 183.9 ± 7.8 |
| Sielnica | 48.861302-18.530103 | 7.6.2025 14:35 |  | 9.6.2025 | 3090 | 1 | 5.8 | 12.0 | 163.6 ± 9.5 |
| Zbojnicka | 48.2293806-17.1231061 | 18.6.2025 9:05 |  | 18.6.2025 | 3060 | 1 | 10.1 | 6.9 | 315.7 ± 18.4 |
| Diery | 49.25264-19.07216 | 13.6.2025 12:17 |  | 16.6.2025 | 3130 | 1 | 8.0 | 1.8 | 11.5 ± 1.5 |
| Dolnanska | 48.8485581-18.0836342 | 20.6.2025 10:45 |  | 23.6.2025 | 3200 | 2 | 12.5 | 0.8 | 85.1 ± 7.5 |
| Frankovka | 48.3281056-17.2053503 | 17.6.2025 10:33 |  | 17.6.2025 | 3060 | 1 | 9.2 | 18.0 | 13.4 ± 1.7 |
| Chleb | 49.1810019-19.0496897 | 12.6.2025 11:22 |  | 16.6.2025 | 3095 | 1 | 5.0 | 10.0 | 27.7 ± 2.6 |
| Kada | 49.0430119-19.677984 | 13.4.2025 12:28 |  | 14.-15.4.2025 | 3020 | 4 | 20.0 | - | 2.7 ± 0.7 |
| Kostolany | 48.41496896-18.2458056 | 13.5.2025 17:00 |  | 14.5.2025 | 2880 | 1 | 10.0 | <15 | 14.2 ± 1.2 |
| Patrovce | 48.779336-18.091183 | 22.4.2025 12:00 |  | 23.4.2025 | 3050 | 1 | 8.8 | 6.0 | 33.8 ± 1.9 |
| Bojnice | 48.775388-18.571156 | 7.6.2025 16:00 |  | 9.6.2025 | 3110 | 1 | 35.6 | 0.8 | 3.5 ± 0.7 |
| Kalameny | 49.13337-19.41969 | 13.4.2025 11:43 |  | 15.4.2025 | 2700 | 2 | 33.0 | - | 9.0 ± 1.1 |
| Kovacova | 48.6079042-19.100739 | 3.5.2025 11:00 |  | 5.5.2025 | 3200 | 1 | 49.0 | 2.1 | 18.4 ± 1.1 |
| Lucky | 49.1342418-19.3972551 | 27.4.2025 13:06 |  | 29.4.2025 | 3090 | 1 | 35.2 | - | 13.1 ± 1.3 |
| Piestany1 | 48.5869165-17.842682 | 10.6.2025 10:25 |  | 9.6.2025 | 3060 | 1 | 48.8 | 2.0 | 68.0 ± 4.6 |
| Piestany2 | 48.586913-17.841779 | 10.6.2025 10:30 |  | 9.6.2025 | 3110 | 1 | 55.5 | 3.6 | 56.5 ± 3.7 |
| Piestany3 | 48.5856106-17.8446239 | 10.6.2025 10:40 |  | 9.6.2025 | 3050 | 1 | 68.0 | 10.0 | 28.0 ± 2.2 |
| Rajec | 49.1292839-18.6821406 | 10.5.2025 13:58 |  | 12.5.2025 | 3060 | 1 | 35.9 | 2.0 | 4.5 ± 0.8 |
| Teplice | 48.909149-18.171703 | 16.5.2025 11:07 |  | 19.5.2025 | 2560 | 1 | 31.2 | 2.3 | 12.0 ± 1.5 |

^a^ Spring name.

Yellowed name - radioactivity exceeding 100 Bq/L, i.e. recommended concentration according to WHO, i.e., Management of radioactivity in drinking-water (https://www.who.int/publications/i/item/9789241513746?utm_source=chatgpt.com).

Blue font, water temperature up to 20 ° C (cold spring),

Red font - water temperature above 30 ° C (temperate and warm springs).

**Table S2.** The distribution of individual archaeal lipids, comprising both core lipids and IPLs identified in samples from 21 springs.

| Name | [M+H]^+^ |
| --- | --- |
| AR | 653.6812 |
| OH-AR | 669.6761 |
| 2OH-AR | 685.6711 |
| PA-AR | 733.6470 |
| PA-OH-AR | 747.6273 |
| PE-AR | 776.6892 |
| PME-AR | 790.7048 |
| PE-OH-AR | 792.6841 |
| **DME-AR^a^** | **804.7205** |
| PME-OH-AR | 806.6997 |
| PG-AR | 807.6837 |
| PS-Uns(5)-AR | 810.6007 |
| Hex-AR | 815.7341 |
| PS-Uns(2)-AR | 816.6477 |
| PG-Uns(2)-OH-AR | 819.6473 |
| PS-AR | 820.6790 |
| **DME-OH-AR** | **820.7154** |
| PG-OH-AR | 823.6786 |
| PS-Uns(2)-OH-AR | 832.6426 |
| PS-OH-AR | 836.6739 |
| PG-diOH-AR | 839.6736 |
| Hex-OH-AR | 848.7549 |
| PS-Uns(1)-diOH-AR | 850.6532 |
| PS-diOH-AR | 852.6688 |
| PG-Uns(1)-Ext-OH-AR | 891.7412 |
| PI-AR | 895.6998 |
| PS-Ext-OH-AR | 906.7521 |
| PI-Uns(2)-OH-AR | 907.6634 |
| PI-OH-AR | 911.6947 |
| PI-diOH-AR | 927.6896 |
| PI-Ext-Uns(5)-OH-AR | 971.6947 |
| 2Hex-AR | 977.7869 |
| PI-Ext-Uns(1)-OH-AR | 979.7573 |
| PI-Ext-OH-AR | 981.7729 |
| PI-Ext-Uns(4)-diOH-AR | 989.7053 |
| 2Hex-Uns(1)-AR | 992.7972 |
| 2Hex-OH-AR | 993.7817 |
| Hex-PS-OH-AR | 998.7267 |
| 3Hex-Uns(1)-AR | 1154.8500 |
| 3Hex-AR | 1156.8656 |
| GDGT-Uns(5*) | 1292.2444 |
| GDGT-Uns(3) | 1296.2757 |
| GDGT-Uns(2) | 1298.2914 |
| GDGT-Uns(1) | 1300.3070 |
| GDGT | 1302.3227 |
| OH-GDGT-Uns(5*) | 1308.2393 |
| OH-GDGT-Uns(3) | 1312.2706 |
| OH-GDGT-Uns(2) | 1314.2863 |
| OH-GDGT-Uns(1) | 1316.3019 |
| OH-GDGT | 1318.3176 |
| diOH-GDGT-Uns(5*) | 1324.2342 |
| diOH-GDGT | 1334.3125 |
| PG-GDGT-Uns(3) | 1450.2788 |
| PG-GDGT-Uns(2) | 1452.2945 |
| Hex-GDGT-Uns(5*) | 1454.2972 |
| PG-GDGT-Uns(1) | 1454.3101 |
| Hex-GDGT-Uns(4) | 1456.3129 |
| PG-GDGT | 1456.3258 |
| Hex-GDGT-Uns(3) | 1458.3286 |
| Hex-GDGT-Uns(2) | 1460.3442 |
| Hex-GDGT-Uns(1) | 1462.3599 |
| Hex-GDGT | 1464.3756 |
| Hex-P-GDGT-Uns(2) | 1540.3105 |
| Hex-P-GDGT-Uns(1) | 1542.3262 |
| Hex-P-GDGT | 1544.3417 |
| diPG-GDGT-Uns(3) | 1604.2819 |
| diPG-GDGT-Uns(2) | 1606.2976 |
| diPG-GDGT-Uns(1) | 1608.3132 |
| diPG-GDGT | 1610.3289 |
| 2Hex-GDGT-Uns(5*) | 1616.3507 |
| 2Hex-GDGT-Uns(4) | 1618.3658 |
| 2Hex-GDGT-Uns(3) | 1620.3815 |
| 2Hex-GDGT-Uns(2) | 1622.3971 |
| 2Hex-GDGT-Uns(1) | 1624.4127 |
| 2Hex-GDGT | 1626.4283 |
| 2Hex-P-GDGT-Uns(5*) | 1696.3166 |
| 2Hex-P-GDGT-Uns(4) | 1698.3322 |
| 2Hex-P-GDGT-Uns(3) | 1700.3477 |
| 2Hex-P-GDGT-Uns(2) | 1702.3633 |
| 2Hex-P-GDGT-Uns(1) | 1704.3790 |
| 2Hex-P-GDGT | 1706.3946 |
| PG-2Hex-GDGT-Uns(3) | 1774.3845 |
| PG-2Hex-GDGT-Uns(2) | 1776.4001 |
| 3Hex-GDGT-Uns(5*) | 1778.4029 |
| PG-2Hex-GDGT-Uns(1) | 1778.4158 |
| 3Hex-GDGT-Uns(4) | 1780.4185 |
| PG-2Hex-GDGT | 1780.4314 |
| 3Hex-GDGT-Uns(3) | 1782.4342 |
| 3Hex-GDGT-Uns(2) | 1784.4499 |
| 3Hex-GDGT-Uns(1) | 1786.4655 |
| 3Hex-GDGT | 1788.4812 |
| 3Hex-P-GDGT-Uns(5*) | 1858.3695 |
| 3Hex-P-GDGT-Uns(3) | 1862.4007 |
| 3Hex-P-GDGT-Uns(2) | 1864.4163 |
| 3Hex-P-GDGT-Uns(1) | 1866.4319 |
| 3Hex-P-GDGT | 1868.4474 |
| 4Hex-GDGT-Uns(5*) | 1940.4560 |
| 4Hex-GDGT-Uns(4) | 1942.4716 |
| 4Hex-GDGT-Uns(3) | 1944.4872 |
| 4Hex-GDGT-Uns(2) | 1946.5027 |
| 4Hex-GDGT-Uns(1) | 1948.5184 |
| 4Hex-GDGT | 1950.5340 |

^a^ **bold**, previously undescribed molecular species, both in nature and synthesized in the laboratory.

**Table S3.** The content of individual molecular species of core lipids in ng/L in 21 springs, determined by shotgun lipidomic.

| **Spring^a^** | **AR^b^** | **OH-AR** | **2OH-AR** | **GDGT** | **OH-GDGT** | **diOH-GDGT** |
| --- | --- | --- | --- | --- | --- | --- |
| Dastinka | 44.8 | 22.7 | 29.2 | 434.0 | 374.5 | 392.0 |
| Emily | 65.6 | 56.5 | 47.4 | 490.0 | 465.5 | 441.0 |
| Himligarka | 51.3 | 31.8 | 43.5 | 451.5 | 399.0 | 430.5 |
| Sielnica | 40.9 | 51.3 | 34.4 | 423.5 | 451.5 | 406.0 |
| Zbojnicka | 57.8 | 53.9 | 59.1 | 469.0 | 458.5 | 472.5 |
| Diery | 155.9 | 166.1 | 104.9 | 297.5 | 318.5 | 192.5 |
| Dolnanska | 150.8 | 118.5 | 132.1 | 287.0 | 220.5 | 248.5 |
| Frankovka | 193.3 | 94.7 | 89.6 | 374.5 | 171.5 | 161.0 |
| Chleb | 118.5 | 96.4 | 103.2 | 220.5 | 175.0 | 189.0 |
| Kada | 174.6 | 69.2 | 84.5 | 336.0 | 119.0 | 150.5 |
| Kostolany | 196.7 | 123.6 | 99.8 | 381.5 | 231.0 | 182.0 |
| Patrovce | 111.7 | 101.5 | 89.6 | 206.5 | 185.5 | 161.0 |
| Bojnice | 266.1 | 287.1 | 278.7 | 22.4 | 36.4 | 30.8 |
| Kalameny | 272.4 | 276.6 | 303.9 | 26.6 | 29.4 | 47.6 |
| Kovacova | 272.4 | 282.9 | 270.3 | 26.6 | 33.6 | 25.2 |
| Lucky | 285.0 | 320.7 | 295.5 | 35.0 | 58.8 | 42.0 |
| Piestany 1 | 293.4 | 293.4 | 310.2 | 40.6 | 40.6 | 51.8 |
| Piestany 2 | 335.4 | 364.8 | 322.8 | 68.6 | 88.2 | 60.2 |
| Piestany 3 | 266.1 | 287.1 | 278.7 | 22.4 | 36.4 | 30.8 |
| Rajec | 272.4 | 276.6 | 303.9 | 26.6 | 29.4 | 47.6 |
| Teplice | 272.4 | 282.9 | 270.3 | 26.6 | 33.6 | 25.2 |

^a^ Spring name in:

Blue font, water temperature up to 20 ° C (cold spring),

Red font - water temperature above 30 °C (temperate and warm springs),

Yellowed name - radioactivity exceeding 100 Bq/L, i.e. recommended concentration according to WHO.

^b^ Abbreviations: AR - archaeol (C20-C20 isoprenoidal chains); OH-AR - monohydroxylated-archaeol; 2OH-AR - dihydroxylated-archaeol; glycerol dialkyl glycerol tetraether - GDGT; monohydroxylated glycerol dialkyl glycerol tetraether - OH-GDGT; diOH-GDGT - dihydroxylated glycerol dialkyl glycerol tetraether.

**Table S4.** The content of individual molecular species of glycosides in ng/L in 21 springs, determined by shotgun lipidomic.

| **Spring^a^** | **Hex-AR^a^** | **2Hex-AR** | **3Hex-AR** | **Hex-GDGT** | **2Hex-GDGT** | **3Hex-GDGT** | **4Hex-GDGT** |
| --- | --- | --- | --- | --- | --- | --- | --- |
| Dastinka | 6.5 | 7.7 | 7.2 | 271.3 | 254.3 | 330.5 | 197.9 |
| Emily | 3.8 | 7.1 | 8.5 | 297.6 | 333.0 | 274.0 | 245.9 |
| Himligarka | 10.2 | 11.7 | 9.5 | 296.7 | 278.1 | 299.5 | 210.1 |
| Sielnica | 7.9 | 7.9 | 8.9 | 265.8 | 321.8 | 288.4 | 242.6 |
| Zbojnicka | 13.0 | 7.5 | 6.7 | 300.9 | 343.5 | 316.7 | 251.4 |
| Diery | 114.2 | 92.6 | 128.0 | 191.3 | 181.4 | 144.4 | 185.5 |
| Dolnanska | 112.1 | 130.1 | 125.1 | 197.9 | 245.9 | 251.4 | 186.8 |
| Frankovka | 108.9 | 136.0 | 122.3 | 116.6 | 129.6 | 160.3 | 112.7 |
| Chleb | 106.0 | 90.6 | 110.7 | 162.9 | 150.0 | 109.9 | 120.1 |
| Kada | 121.6 | 98.3 | 130.4 | 109.5 | 111.9 | 123.7 | 125.6 |
| Kostolany | 98.5 | 111.1 | 117.0 | 124.9 | 130.8 | 140.2 | 155.8 |
| Patrovce | 106.3 | 100.1 | 123.9 | 252.7 | 230.8 | 228.1 | 174.2 |
| Bojnice | 165.8 | 187.8 | 168.8 | 15.8 | 15.0 | 11.6 | 21.3 |
| Kalameny | 167.9 | 144.2 | 146.3 | 9.0 | 7.6 | 3.4 | 14.3 |
| Kovacova | 186.5 | 195.0 | 120.7 | 16.2 | 15.5 | 12.1 | 16.9 |
| Lucky | 174.1 | 187.6 | 152.7 | 9.9 | 8.6 | 4.5 | 11.5 |
| Piestany1 | 197.7 | 168.9 | 156.0 | 6.5 | 7.7 | 7.2 | 6.9 |
| Piestany 2 | 170.6 | 144.6 | 150.2 | 9.9 | 7.1 | 8.5 | 19.5 |
| Piestany3 | 216.0 | 208.4 | 174.2 | 6.9 | 7.5 | 6.7 | 9.9 |
| Rajec | 148.1 | 133.7 | 210.1 | 7.6 | 9.5 | 8.0 | 8.9 |
| Teplice | 219.9 | 212.8 | 178.9 | 12.8 | 18.7 | 15.1 | 15.6 |

^a,b^ Abbreviations: Hex-AR - glycosyl (hexosyl) archaeol; 2Hex-AR - diglycosyl (dihexosyl) archaeol; 3Hex-AR - triglycosyl (trihexosyl) archaeol; Hex-GDGT - glycosyl (hexosyl) glycerol dialkyl glycerol tetraether; 2Hex-GDGT - diglycosyl (dihexosyl) glycerol dialkyl glycerol tetraether; 3Hex-GDGT - triglycosyl (trihexosyl) glycerol dialkyl glycerol tetraether; 4Hex-GDGT - tetraglycosyl (tetrahexosyl) glycerol dialkyl glycerol tetraether.

**Table S5.** The content of individual molecular species of archaeol phospholipids in ng/L in 21 springs, determined by shotgun lipidomic.

| **Spring^a^** | **PA-AR^b^** | **PE-AR** | **PME-AR** | **DME-AR^c^** | **PG-AR** | **PS-AR** | **PI-AR** |
| --- | --- | --- | --- | --- | --- | --- | --- |
| Dastinka | 164.2 | 171.4 | 108.5 |  | 221.7 | 286.3 | 173.9 |
| Emily | 153.7 | 165.2 | 135.6 |  | 288.4 | 238.4 | 214.6 |
| Himligarka | 148.5 | 128.4 | 130.2 |  | 241.9 | 260.0 | 184.2 |
| Sielnica | 146.7 | 165.3 | 149.2 | 231.4 | 278.9 | 250.6 | 211.8 |
| Zbojnicka | 173.7 | 149.3 | 138.4 |  | 297.3 | 274.6 | 219.2 |
| Diery | 96.3 | 91.0 | 111.2 |  | 186.5 | 157.8 | 163.4 |
| Dolnanska | 150.8 | 128.7 | 133.5 |  | 201.8 | 199.5 | 164.5 |
| Frankovka | 101.2 | 116.4 | 112.2 |  | 159.9 | 128.6 | 101.7 |
| Chleb | 103.0 | 84.7 | 114.7 | 144.2 | 127.8 | 93.3 | 108.0 |
| Kada | 98.5 | 121.4 | 109.8 |  | 133.3 | 99.3 | 112.6 |
| Kostolany | 109.2 | 89.5 | 116.7 |  | 182.8 | 153.8 | 138.2 |
| Patrovce | 89.7 | 100.3 | 105.3 |  | 119.5 | 84.2 | 153.8 |
| Bojnice | 67.3 | 54.9 | 60.0 |  | 12.7 | 12.3 | 24.2 |
| Kalameny | 36.7 | 42.9 | 51.2 |  | 12.2 | 13.4 | 18.3 |
| Kovacova | 68.8 | 50.4 | 8.1 |  | 12.5 | 11.9 | 20.5 |
| Lucky | 63.4 | 44.5 | 28.0 |  | 14.2 | 13.0 | 15.9 |
| Piestany 1 | 52.3 | 32.3 | 14.6 |  | 12.9 | 13.7 | 12.0 |
| Piestany 2 | 73.0 | 55.0 | 39.6 |  | 16.1 | 14.2 | 22.7 |
| Piestany 3 | 46.6 | 25.9 | 7.6 |  | 12.7 | 12.3 | 14.6 |
| Rajec | 74.5 | 56.7 | 41.5 |  | 12.2 | 13.4 | 13.7 |
| Teplice | 50.0 | 29.7 | 11.8 |  | 12.5 | 11.9 | 19.4 |

^a,b^ Abbreviations: PA-AR - phosphatidic acid archaeol; PE-AR - phosphatidylethanolamine archaeol; PME-AR - phosphatidylmethylethanolamine archaeol; DME-AR - phosphatidyldimethylethanolamine archaeol; PG-AR - phosphatidylglycerol archaeol; PS-AR - phosphatidylserine archaeol; PI-AR - phosphatidylinositol archaeol.

^c^ Identified in only two springs; see text.

**Table S6.** Results of the analysis of the KEGG database (https://www.genome.jp/kegg/pathway.html) showing that more than 30 archaeal species are putatively contained by two key *N*-methyltransferases (EC 2.1.1.17 and EC 2.1.1.71).

| **Kegg code** | **Genus** | **Species** | **Strain** |
| --- | --- | --- | --- |
| [hje](https://www.kegg.jp/kegg-bin/show_organism?org=hje) | *Halalkalicoccus* | *jeotgali* |  |
| [harc](https://www.kegg.jp/kegg-bin/show_organism?org=harc) | *Halococcoides* | *cellulosivorans* |  |
| [haln](https://www.kegg.jp/kegg-bin/show_organism?org=haln) | *Halolamina* | sp. | CBA1230 |
| [hah](https://www.kegg.jp/kegg-bin/show_organism?org=hah) | *Halophilic* | *archaeon* |  |
| [hxa](https://www.kegg.jp/kegg-bin/show_organism?org=hxa) | *Halopiger* | *xanaduensis* |  |
| [hwc](https://www.kegg.jp/kegg-bin/show_organism?org=hwc) | *Haloquadratum* | *walsbyi* | C23 |
| [hezz](https://www.kegg.jp/kegg-bin/show_organism?org=hezz) | *Halorubrum* | *ezzemoulense* |  |
| [hss](https://www.kegg.jp/kegg-bin/show_organism?org=hss) | *Halorubrum* | *ruber* |  |
| [halb](https://www.kegg.jp/kegg-bin/show_organism?org=halb) | *Halorubrum* | sp. | BOL3-1 |
| [hlt](https://www.kegg.jp/kegg-bin/show_organism?org=hlt) | *Halosimplex* | *litoreum* |  |
| [hpel](https://www.kegg.jp/kegg-bin/show_organism?org=hpel) | *Halosimplex* | *pelagicum* |  |
| [hrr](https://www.kegg.jp/kegg-bin/show_organism?org=hrr) | *Halosimplex* | *rubrum* |  |
| [hlr](https://www.kegg.jp/kegg-bin/show_organism?org=hlr) | *Halostagnicola* | *larsenii* |  |
| [hakz](https://www.kegg.jp/kegg-bin/show_organism?org=hakz) | *Haloterrigena* | *alkaliphila* |  |
| [hsal](https://www.kegg.jp/kegg-bin/show_organism?org=hsal) | *Haloterrigena* | *salifodinae* |  |
| [htu](https://www.kegg.jp/kegg-bin/show_organism?org=htu) | *Haloterrigena* | *turkmenica* |  |
| [mefw](https://www.kegg.jp/kegg-bin/show_organism?org=mefw) | *Methanochimaera* | *problematica* |  |
| [naj](https://www.kegg.jp/kegg-bin/show_organism?org=naj) | *Natrarchaeobaculum* | *aegyptiacum* |  |
| [nag](https://www.kegg.jp/kegg-bin/show_organism?org=nag) | *Natrarchaeobaculum* | *sulfurireducens* | AArc-Mg |
| [nan](https://www.kegg.jp/kegg-bin/show_organism?org=nan) | *Natrarchaeobaculum* | *sulfurireducens* | AArc1 |
| [nmg](https://www.kegg.jp/kegg-bin/show_organism?org=nmg) | *Natrialba* | *magadii* |  |
| [haly](https://www.kegg.jp/kegg-bin/show_organism?org=haly) | *Natrinema* | *halophilum* |  |
| [hlo](https://www.kegg.jp/kegg-bin/show_organism?org=hlo) | *Natrinema* | *longum* |  |
| [npe](https://www.kegg.jp/kegg-bin/show_organism?org=npe) | *Natrinema* | *pellirubrum* |  |
| [hjt](https://www.kegg.jp/kegg-bin/show_organism?org=hjt) | *Natrinema* | *thermotolerans* |  |
| [nvr](https://www.kegg.jp/kegg-bin/show_organism?org=nvr) | *Natrinema* | *versiforme* |  |
| [nge](https://www.kegg.jp/kegg-bin/show_organism?org=nge) | *Natronobacterium* | *gregoryi* |  |
| [hlc](https://www.kegg.jp/kegg-bin/show_organism?org=hlc) | *Natronobacterium* | *lacisalsi* |  |
| [nou](https://www.kegg.jp/kegg-bin/show_organism?org=nou) | *Natronococcus* | *occultus* |  |
| [nho](https://www.kegg.jp/kegg-bin/show_organism?org=nho) | *Natronomonas* | *halophila* |  |
| [nas](https://www.kegg.jp/kegg-bin/show_organism?org=nas) | *Natronorubrum* | *aibiense* |  |
| [nbg](https://www.kegg.jp/kegg-bin/show_organism?org=nbg) | *Natronorubrum* | *bangense* |  |
| [hda](https://www.kegg.jp/kegg-bin/show_organism?org=hda) | *Natronorubrum* | *daqingense* |  |
| [sali](https://www.kegg.jp/kegg-bin/show_organism?org=sali) | *Salinarchaeum* | sp. | Harcht-Bsk1 |

**Table S7.** Variable Importance in Projection (VIP) scores and PLS loading values for lipids along the radon-activity and temperature gradients derived from the first two PLS components.

| Lipid Compound | VIP score | PLS loading – radon gradient | PLS loading – temperature gradient |
| --- | --- | --- | --- |
| AR | 0.876 | -0.197 | -0.114 |
| OH-AR | 1.109 | -0.193 | -0.436 |
| 2OH-AR | 1.180 | -0.185 | -0.478 |
| GDGT | 0.906 | 0.355 | 0.270 |
| OH-GDGT | 0.972 | 0.372 | -0.290 |
| diOH-GDGT | 1.141 | 0.386 | -0.451 |
| Hex-AR | 0.942 | -0.160 | 0.048 |
| 2Hex-AR | 0.963 | -0.156 | 0.092 |
| 3Hex-AR | 1.297 | -0.156 | 0.278 |
| Hex-GDGT | 0.900 | 0.250 | 0.058 |
| 2Hex-GDGT | 0.924 | 0.277 | -0.026 |
| 3Hex-GDGT | 0.875 | 0.267 | -0.015 |
| 4Hex-GDGT | 0.887 | 0.188 | 0.143 |
| PA-AR | 0.955 | 0.095 | -0.061 |
| PE-AR | 0.866 | 0.099 | 0.017 |
| PME-AR | 1.322 | 0.078 | 0.255 |
| PG-AR | 0.858 | 0.227 | 0.057 |
| PS-AR | 0.948 | 0.231 | -0.096 |
| PI-AR | 0.887 | 0.160 | 0.122 |


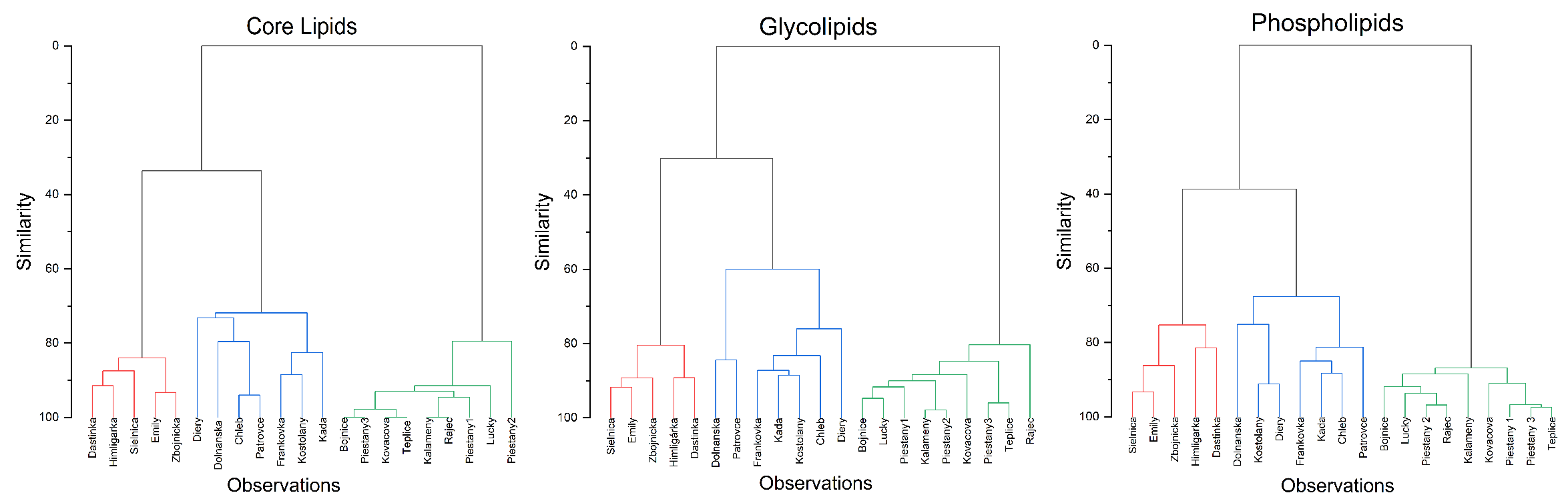


**Fig. S1.** Hierarchical cluster analysis (HCA) dendrograms of core lipids, glycolipids, and phospholipids, indicating three main clusters of springs.

*
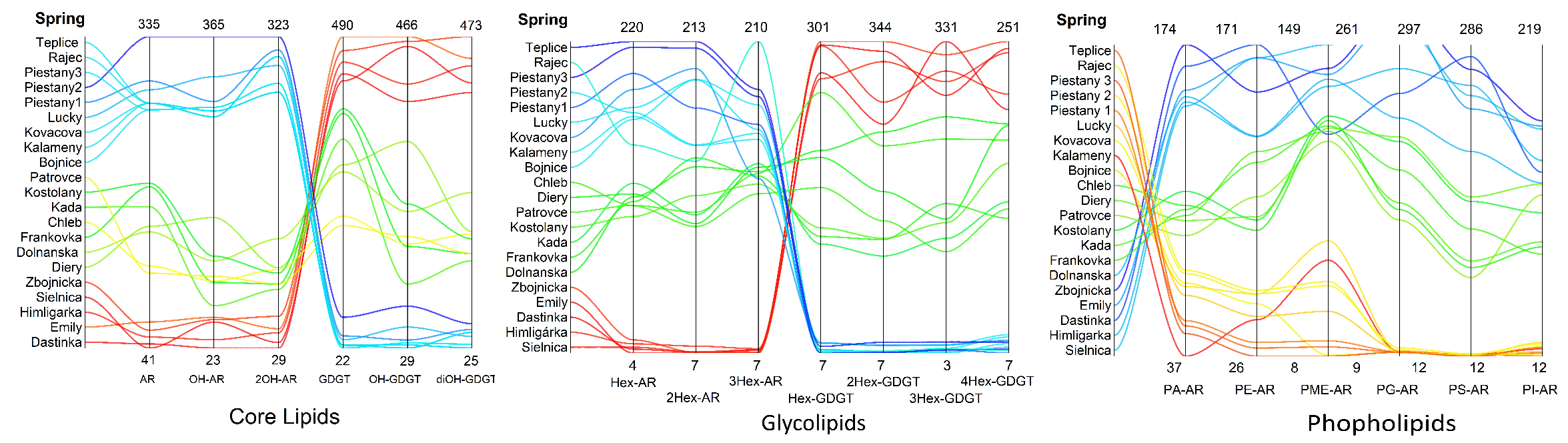
*

**Fig. S2.** Relative abundances of individual archaeal lipid biomarkers in the investigated springs, grouped by lipid class. Springs with similar lipid profiles may appear in similar shades, which can visually resemble sample grouping.

**Fig. S3.** Structure of DMPE-AR, i.e., 2,3-bis((3,7,11,15-tetramethylhexadecyl)oxy)propyl (2-(dimethylamino)ethyl) hydrogen phosphate.


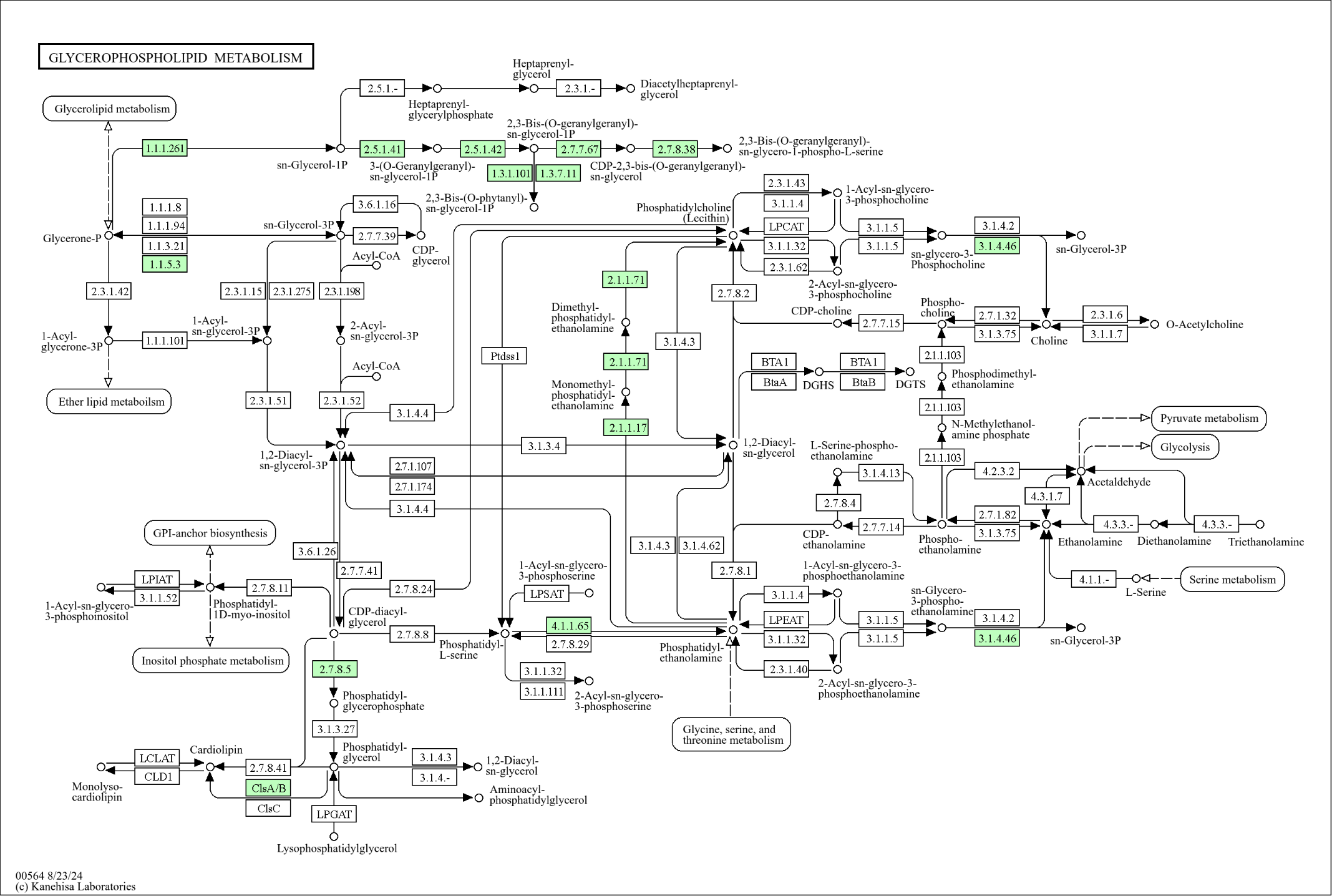


**Fig. S4**. Kegg *Haloquadratum walsbyi*. DSM 16790 ano


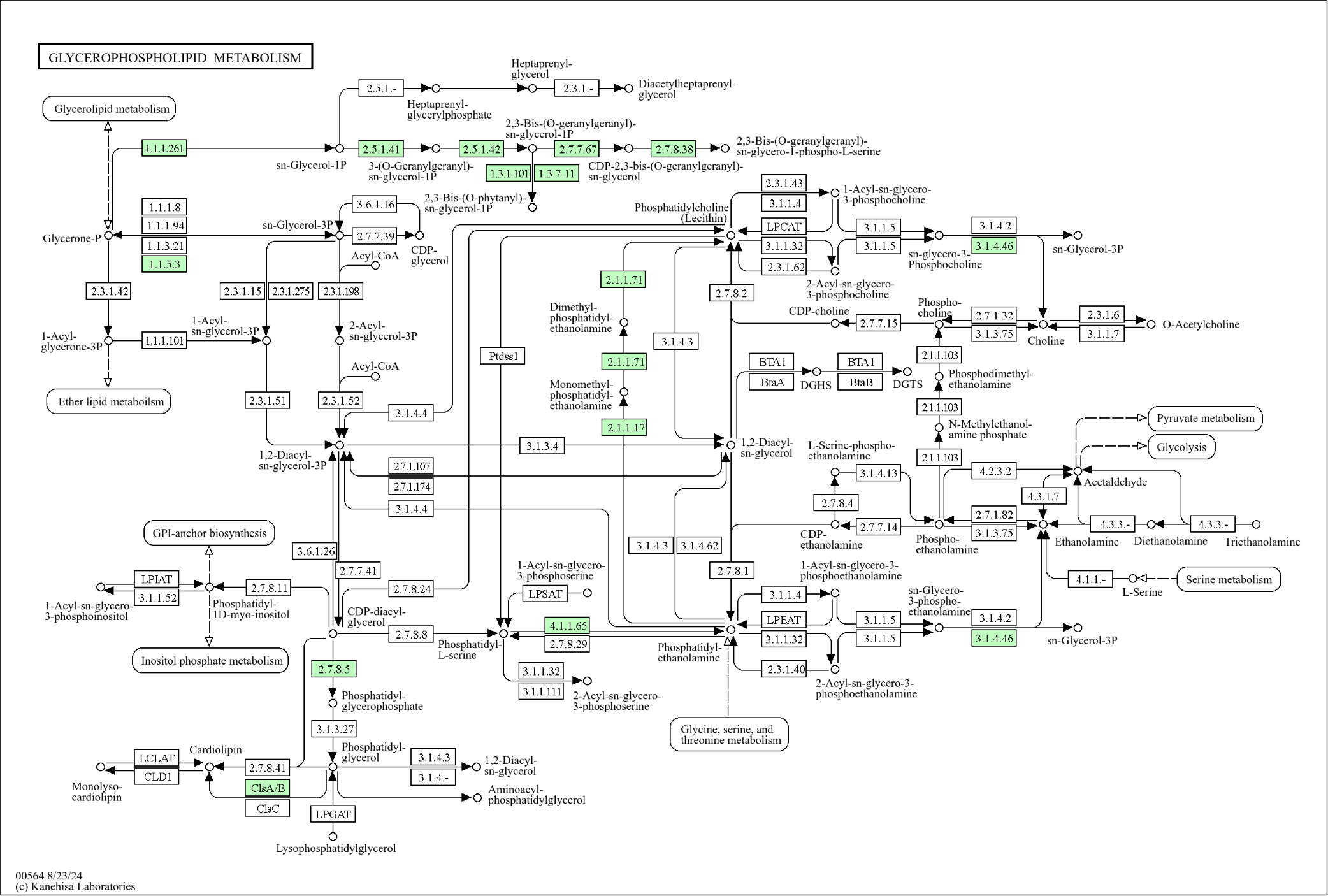


**Fig. S5.** Kegg *Natrarchaeobaculum sulfurireducens*. AArc1 (strain) ano


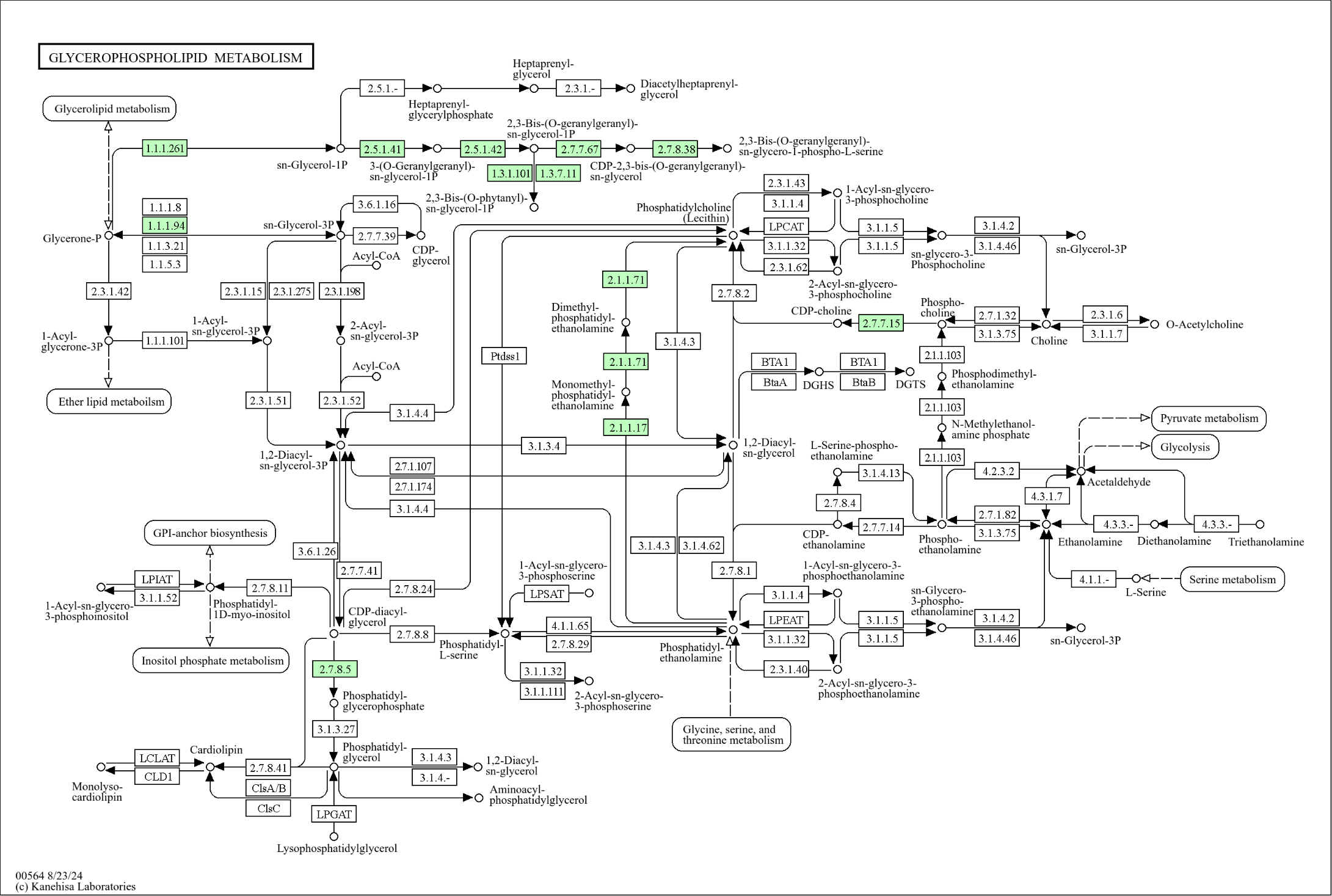


**Fig. S6.** Kegg *Methanobrevibacter smithii*. DSM 11975 ano
